# Significant health impact of US small turtle-related Salmonella outbreak

**DOI:** 10.1016/j.nmni.2023.101173

**Published:** 2023-09-08

**Authors:** Emmanuel Kokori, Gbolahan Olatunji, Adeola Akinboade, Aminat Akinoso Ayomide, Bolakale Akanbi II M-T, Khalimat Moradeyo Temitope, Emmanuel Egbunu, Nicholas Aderinto

**Affiliations:** Department of Medicine and Surgery, University of Ilorin, Kwara State, Nigeria; Federal Medical Centre, Bida, Nigeria; Department of Medicine, Ladoke Akintola University of Technology, Nigeria

**Keywords:** Salmonella outbreak, Small turtles, Health impact, Disease transmission, Zoonotic disease

Dear Editor,

This letter discusses a recent Salmonella outbreak linked to small turtles in the United States, affecting 26 individuals, with the first case reported on October 27, 2022 [1]. The current outbreak is associated with Salmonella Stanley (24 cases) and Salmonella Pomona (2 cases), spanning 11 U.S. states [1]. Children under 5 years old are the most susceptible, with 11 cases reported in this age group [1]. Symptoms range from fever to abdominal cramps, with most individuals recovering within a week [1]. The aim is to shed light on this outbreak, considering the possibility of unreported cases. Emphasis is placed on the persistent health risks posed by small turtles as carriers of Salmonella despite federal regulations.

The association between Salmonellosis and small turtles dates back to the 1960s when small turtles gained popularity as pets in the United States [1]. By the 1970s, 15% of Salmonella infections were linked to small turtles, leading to a federal ban in 1975 on interstate sales and distribution of turtles less than 4 inches [1]. However, this ban has not eliminated the problem, as small turtles continue to be kept as pets, posing a substantial risk due to their role as reservoirs for Salmonella.

Salmonella, named after American Veterinary surgeon Elmer Salmon, is a gram-negative bacillus bacterium with over 2600 serotypes [2]. It causes approximately 1.2 million cases and 450 fatalities annually in the United States [2]. While Salmonella is primarily transmitted feco-orally between humans, non-typhoidal Salmonella is a zoonotic disease, as exemplified by this outbreak.

Zoonotic diseases, transmitted between vertebrate animals and humans, are a global concern. Over 60% of known infectious diseases in humans are spread from animals, and 75% of emerging infectious diseases stem from animals [3]. They can be transmitted directly or indirectly through various modes, including direct contact, indirect contact, vector-borne transmission, foodborne transmission, and waterborne transmission [4].

Zoonotic diseases pose substantial economic burdens due to losses in agricultural production, prevention and treatment costs, and decreased productivity. Understanding and effectively managing zoonotic diseases are vital to safeguarding public health and economic stability.

U.S. health authorities have implemented various measures to combat the recent Salmonella outbreak, including epidemiological investigations, advisories, new regulations, collaboration, and monitoring [5]. While these efforts have been moderately effective, challenges such as identifying outbreak sources, communicating with the public, and evaluating the response's impact persist.

This outbreak underscores the need for a broader public health perspective that considers the interconnectedness of human, animal, and environmental health—a concept known as the “One Health” approach. It highlights the importance of educating pet owners about the health risks associated with various animals. It calls for tighter controls on pet sales, especially to vulnerable populations like young children and the elderly.

The recent Salmonella outbreak linked to small turtles is a stark reminder of the complex relationships between humans, animals, and the environment. It urges interdisciplinary collaboration, improved policies, and heightened public awareness to address zoonotic diseases effectively. Furthermore, it emphasises the profound impact of human choices and behaviours on ecological stability, underscoring the need for a harmonious coexistence prioritising all species' health and well-being.

## Data statement

No new datasets were generated for this paper.

## Funding

No funding was received for this paper.

## Declaration of competing interest

The authors declare no conflict of interest.
